# Biochemical Validation of the Older Australian’s Food Frequency Questionnaire Using Carotenoids and Vitamin E

**DOI:** 10.3390/nu6114906

**Published:** 2014-11-06

**Authors:** Jun S. Lai, John Attia, Mark McEvoy, Alexis J. Hure

**Affiliations:** 1 School of Medicine and Public Health, University of Newcastle, New South Wales 2308, Australia; E-Mails: john.attia@newcastle.edu.au (J.A.); mark.mcevoy@newcastle.edu.au (M.M.); alexis.hure@newcastle.edu.au (A.J.H.); 2 Hunter Medical Research Institute, New Lambton Heights, New South Wales 2305, Australia; 3 John Hunter Hospital, New Lambton Heights, New South Wales 2305, Australia

**Keywords:** validation, food frequency questionnaire, biomarkers, carotenoids, vitamin E

## Abstract

Background: Validation of a food frequency questionnaire (FFQ) is important, as inaccurate and imprecise information may affect the association between dietary exposure and health outcomes. Objective: This study assessed the validity of the Older Australian’s FFQ against plasma carotenoids and Vitamin E. Methods: A random subsample (*n* = 150) of 2420 participants in the Hunter Community Study, aged 55–85 years, were included. Correlations between crude and energy-adjusted FFQ estimates of carotenoids, Vitamin E, and fruit and vegetables with corresponding biomarkers were determined. Percentages of participants correctly classified in the same quartile, and in the same ± 1 quartile, by the two methods were calculated. Results: Significant correlations (*P* < 0.05) were observed for α-carotene (*r* = 0.26–0.28), β-carotene (*r* = 0.21–0.25), and β-cryptoxanthin (*r* = 0.21–0.23). Intakes of fruits and vegetables also showed similar correlations with these plasma carotenoids. Lycopene was only significantly correlated with fruit and vegetable intakes (*r* = 0.19–0.23). Weak correlations were observed for lutein + zeaxanthin (*r* = 0.12–0.16). For Vitamin E, significant correlation was observed for energy-adjusted FFQ estimate and biomarker (*r* = 0.20). More than 68% of individuals were correctly classified within the same or adjacent quartile, except for lutein + zeaxanthin. Conclusion: With the exception of lutein + zeaxanthin, the Older Australian’s FFQ provides reasonable rankings for individuals according to their carotenoids, Vitamin E, fruit and vegetable intakes.

## 1. Introduction

A number of methods are used to measure dietary intake in epidemiological research including dietary recalls, food records, and food frequency questionnaires (FFQs) [1]. Of these, dietary recalls and food records are considered more precise, but they are limited in that they only measure short-term dietary intake. However, FFQs provide dietary data over a longer period of time [1], which in nutritional epidemiologic research is more important than intake on a few specific days. A number of FFQs have been developed to measure dietary intake among Australian adults [2,3,4]. Considering the fact that older people may differ in dietary habits and food patterns from younger adults [5], existing FFQs should be adapted to reflect these differences, and/or validated in older populations. 

FFQs are often criticised for having a large number of measurement errors [1]. Consequently, much research has been concerned with the relative performance of FFQs in estimating dietary intake. Most studies have validated FFQs against food records or dietary recall [2,3], but self-reporting bias remains. Alternatively, biochemical indicators (or biomarkers) can act as objective measures in the validation of nutrient intake, as the errors recorded are assumed to be independent of self-report [1]. The body of literature on the performance of FFQs in older populations is relatively small, and validation against biochemical indicators is scarce. To date, we identified only one FFQ, developed by the Blue Mountains Eye Study (BMES), to measure dietary intake among older community-dwelling adults in Australia, which has been previously validated against 4-day food records [3]. In this study, we will further assess the validity of this FFQ against more objective biochemical indicators, using a sub-population of older Australian adults from the Hunter Community Study (HCS). 

With increasing evidence that high intakes of fruits and vegetables are associated with better health outcomes [6,7], it is important that the FFQ used adequately captures these foods among the population of interest. The protective effects of fruit and vegetables may be due to their antioxidant properties. Nutrients such as carotenoids and Vitamin E have the ability to reduce inflammation and prevent free radical damage, all of which have been shown to play important functions in the biology of ageing [8,9]. Furthermore, concentrations of carotenoids and Vitamin E in blood are considered reliable markers of dietary intake [1] and have been previously used in a number of dietary validation studies [10,11,12]. Hence, this study aims to compare the dietary intakes of carotenoids and Vitamin E, estimated by Older Australian’s FFQs, against plasma biomarkers, in a sample of 150 HCS participants.

## 2. Methods

### 2.1. Validation Quality

This study was developed based on the EURopean micronutrient RECommendations Aligned Network of Excellence (EURRECA) scoring system of a good quality validation study [13], scoring a total of 4 out of 7 points. The allocation of points was as follows: (1) 0.5 points for non-homogenous sample, and 0.5 points for a sample size of >50; (2) 1.5 points for reporting crude and energy-adjusted correlation coefficients, and including statistics to assess classification; and (3) 1.5 points for including supplements intake. 

### 2.2. Subjects

The study subjects were drawn from the HCS. The HCS is a population based cohort study of adults aged 55–85 years residing in Newcastle, New South Wales state, randomly selected from the state’s electoral roll [14]. Recruitment began in December 2004 and ended in December 2007. A total of 3253 individuals participated in the study. All participants were required to attend a clinical assessment, provide a blood sample and complete a series of self-administered questionnaires including Older Australian’s FFQs [14]. Full methodological details have been published previously [14]. The HCS has received ethics approval from the University of Newcastle Research Ethics Committee (H-820-0504), and all participants provided written informed consent. 

**Figure 1:**
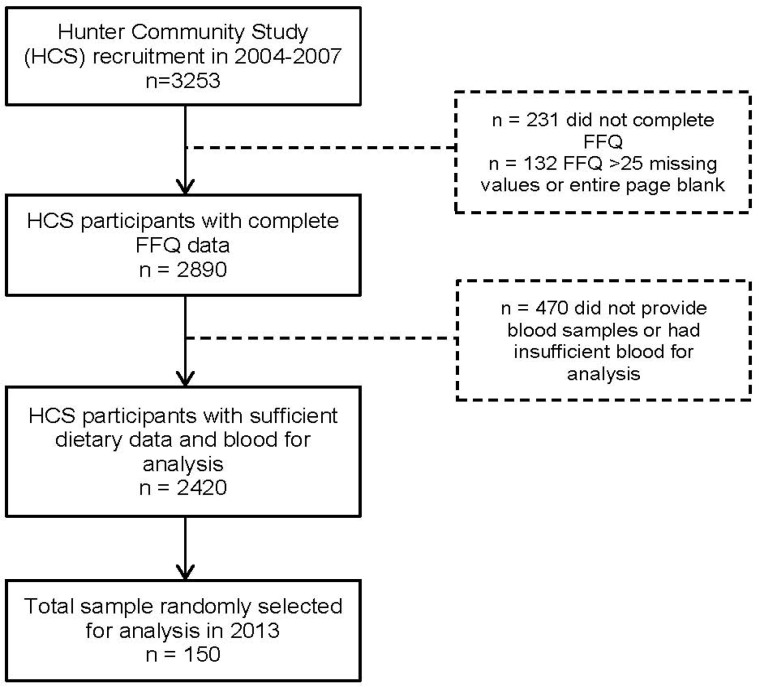
Participant flow diagram for a validation study of food frequency questionnaire (FFQ) estimated intakes against biomarkers.

To be included in this study, participants needed to have completed the FFQ (*n* = 3022) and provided a blood sample at baseline with enough volume for analysis (*n* = 2534). Participants with more than 25 missing values or an entire blank page in their FFQ (*n* = 132) were excluded from the final dataset. A subset of 150 subjects was selected from the remaining 2420 HCS participants. Stratified random sampling using computer-generated sequence was used to select 30 participants from each quintile of total energy intake, and ensuring an equal representation across gender and age groups (<65, 65+). A participant selection flow diagram is presented in Figure 1.

### 2.3. Food Frequency Questionnaire

Dietary intake was assessed by a self-administered, 145-item semi-quantitative FFQ [3], modified from the version developed by Willett [15], specifically for use with older Australians participating in the BMES [3]. The BMES previously validated this FFQ against 4-day weighed food records and demonstrated reasonable validity (*i.e.*, *r* ≥ 0.5 for most nutrients including *r* = 0.49 for β-carotene; and ≥70% correctly classified within same ± 1 quintile) [3]. Participants were required to indicate their usual frequency of foods consumed in the past year, with nine categorical frequency options, ranging from never to four or more times per day. Open-ended questions were included on the type of fruit juices, breakfast cereal, and other frequently consumed foods that were not included in the list. The FFQ also assessed dietary supplement usage. Participants completed the FFQ within three months of their blood collection. Dietary intake of carotenoids and Vitamin E was calculated using the US Department of Agriculture (USDA) data [16], and other nutrient intakes were derived from NUTTAB 2006, an Australian nutrient composition database [17]. Servings of fruits and vegetables were defined based on the Australian Dietary Guidelines (e.g., one serving of fruit = 150 g or 1 medium-sized fruit; one serving of vegetables = 75 g or ½ cup cooked vegetables) [18]. Nutrient supplement information was obtained from manufacturers and added to the database. Approximately 2% of all FFQs were re-entered into the Food Works 2009, version 6 [19], by an Accredited Practicing Dietitian who was blinded to the original FFQ data entry to check for errors. Only minor discrepancies were observed and rectified prior to data analysis. 

### 2.4. Biomarkers Assays

The biomarkers included in this study were plasma concentrations of α-carotene, β-carotene, β-cryptoxanthin, lycopene, lutein + zeaxanthin, and vitamin E (α-tocopherol). The plasma and FFQ estimates of lutein and zeaxanthin are shown combined (lutein + zeaxanthin) because both the nutrient database and biochemical analysis combine lutein and zeaxanthin. Fasting venous blood was obtained using standard venepuncture techniques. All blood samples were centrifuged and stored in approximately 1 mL aliquots, cryopreserved in dimethyl sulfoxide (DMSO) at −80°C immediately after collection [14]. These blood samples had been stored at −80°C for approximately seven years at the time of assay, and were thawed immediately prior to analysis. Plasma carotenoids concentrations were determined using the high performance liquid chromatography method [20]. Measurements of red blood cell (RBC) folate concentration was carried out using the chemiluminescent immunoassay analyser (Access^®^ Immunoassay Systems, Beckman Coulter, Inc., CA, USA) [21]. However, it was subsequently determined that DMSO had likely affected the integrity of RBC membrane, reducing the accuracy of the folate concentration. Therefore, subsequent results and discussion will focus on carotenoids and Vitamin E only. 

### 2.5. Statistical Analysis

Dietary intakes were expressed as absolute amounts and as energy-adjusted intakes. Energy-adjusted intakes were computed for individual carotenoids, Vitamin E, servings of fruits and vegetables, using the residual method [22]. Adjusting for total energy intake accounts for between-person variation in total energy intake as a result of physiological differences such as body size and physical activity [22], thus reducing the potentially confounding effects of total energy intake. As the distribution of dietary intakes and biomarkers were skewed, Spearman rank correlation coefficients were used in all correlation analyses, unless otherwise specified. The significance level was set at *P* < 0.05. Aside from comparing individual dietary intakes of carotenoids and Vitamin E to their respective plasma biomarkers, fruit and vegetable intakes were also compared to each plasma carotenoid. Intakes of fruits and vegetables were restricted to those that contributed ≥5% of daily mean intake for each carotenoid (e.g., carrot and pumpkin intakes to plasma α-carotene). We did not compare intakes of fruits and vegetables to plasma Vitamin E, because Vitamin E comes from more diverse sources than each of the carotenoids. 

Linear regression analyses were performed to identify potential confounders. Plasma carotenoids and Vitamin E were modelled as the dependent variables and the corresponding FFQ estimated intakes as the independent variables. This was performed using log-transformed values to comply with the assumptions for normality. The following variables were tested as potential confounders: age groups, gender, smoking status, body mass index (BMI) categories, medication use, supplement use and alcohol consumption. The significance level was set at *P* < 0.05.

Dietary intakes estimated by the FFQ and biomarkers were classified into quartiles to determine the ability of both methods to rank individuals. Percentages were calculated for participants correctly classified into the same quartile and within the adjacent quartile. All statistical analyses were performed using Stata, version 11 [23]. 

## 3. Results

Characteristics of the sampled subjects, along with their average daily nutrient intake values from the FFQ and measured biomarkers are presented in Table 1. The participants’ ages ranged from 55–85 years. Stratified random sampling ensured a similar proportion of males and females. Mean BMI was 28.5 kg/m^2^ indicating a high proportion of overweight and obesity, which is consistent with the broader Australian population of this age [24]. Only 6% of the sample currently smoked but many more were former smokers (41.3%). A large proportion of the sample (78.6%) was taking at least one prescription medication, which is not surprising for an older population. Approximately 12% of the subjects reported taking supplements containing Vitamin A (including carotenoids) and/or Vitamin E. Approximately 63% of participants reported consuming alcoholic beverages at least once a week. 

Carrots and pumpkins were the main contributors to α-carotene intake. Sources of β-carotene included apricots or peaches, cantaloupe, broccoli, carrots, spinach or silverbeet, lettuce, peas, pumpkin, sweet potato. Intake of β-cryptoxanthin was from paw-paw, orange, pumpkin, apricots or peaches, carrots and corn. Lycopene was predominantly from tomato and tomato products, but also included watermelon and grapefruit. Lutein and zeaxanthin were mainly from dark green vegetables such as broccoli, brussel sprout, spinach or silverbeet, lettuce, and beans, peas, corn and pumpkin. 

**Table 1:** Characteristics of Hunter Community Study participants (*n* = 150) in a dietary validation study.

Characteristics	
Age (years), mean ± SD	66.2 ± 7.3
Gender, *n* (%)	
Male	77 (51.3%)
Female	73 (48.7%)
Body Mass Index, kg/m^2^	28.5 ± 4.8
Smoking status ^a^, *n* (%)	
Non-smoker	73 (48.7%)
Ex-smoker	62 (41.3%)
Current smoker	9 (6%)
Current use of medication, *n* (%)	118 (78.6%)
Supplement use, *n* (%)	
Multivitamin ^b^	15 (10%)
Vitamin E only	3 (2%)
Alcohol intake, *n* (%)	
None	55 (36.7%)
≥1 drink/week	95 (63.3%)
FFQ estimated nutrient intake, mean ± SD	
α-carotene, μg/day	1810 ± 1499
β-carotene, μg/day	8449 ± 5005
β-cryptoxanthin, μg/day	590 ± 372
Lycopene, μg/day	6457 ± 6276
Lutein + zeaxanthin, μg/day	4026 ± 2538
Vitamin E, mg/day	5.8 ± 2.3
FFQ estimated fruit + vegetable intakes, mean ± SD	
α-carotene sources, servings/day	0.8 ± 0.6
β-carotene sources, servings/day	2.8 ± 1.4
β-cryptoxanthin sources, servings/day	2.2 ± 1.3
Lycopene sources, servings/day	0.8 ± 0.6
Lutein + zeaxanthin sources, servings/day	2.2 ± 0.9
Plasma concentration, mean ± SD	
α-carotene, mg/L	0.07 ± 0.06
β-carotene, mg/L	0.35 ± 0.40
β-cryptoxanthin, mg/L	0.14 ± 0.12
Lycopene, mg/L	0.29 ± 0.14
Lutein + zeaxanthin, mg/L	0.47 ± 0.27
Vitamin E, mg/L	13.61 ± 4.08

^a^ *n* = 6 did not report smoking status; ^b^ Multivitamin supplements containing carotenoids and Vitamin E.

Results from linear regression showed that age, gender, smoking status, BMI, medication use, supplement use and alcohol consumption had little effect on correlation coefficients. As such, the correlation coefficients were only reported for crude intakes and energy-adjusted intakes. 

Correlations between FFQ estimated intakes of individual carotenoids, Vitamin E, fruit and vegetables, and plasma concentrations are presented in Table 2. Both crude and adjusted correlations were significant for α-carotene (*r* = 0.26 and 0.28), β-carotene (*r* = 0.21 and 0.25) and β-cryptoxanthin (*r* = 0.21 and 0.23). Energy-adjusted Vitamin E intake yielded a stronger correlation with plasma concentration (*r* = 0.20) compared to crude intake (*r* = 0.08). In contrast, weak correlations were observed for lycopene and lutein + zeaxanthin. Intakes of fruits and vegetables showed significant correlations with plasma α-carotene (*r* = 0.23 and 0.25) and β-carotene (*r* = 0.20 and 0.25), respectively. Interestingly, correlations between fruit and vegetable intakes, and plasma β-cryptoxanthin (*r* = 0.31 and 0.36) and lycopene (*r* = 0.19 and 0.23) were much higher than the corresponding nutrient intakes. Plasma lutein + zeaxanthin was weakly correlated with fruit and vegetable intakes (*r* = 0.11 and 0.14). 

**Table 2:** Correlations and 95% CI between FFQ estimated intakes and biomarkers.

	Individual Nutrient Intakes				Fruit and Vegetable Intakes			
	*r*_crude_^a^	95% CI	*r*_adj_^b^	95% CI	*r*_crude_^a^	95% CI	*r*_adj_^b^	95% CI
α-carotene	0.26 ^c^	0.10, 0.38	0.28 ^c^	0.12, 0.42	0.23 ^c^	0.07, 0.38	0.25 ^c^	0.08, 0.39
β-carotene	0.21 ^d^	0.04, 0.35	0.25 ^c^	0.08, 0.39	0.20 ^d^	0.04, 0.36	0.25 ^c^	0.08, 0.38
β-cryptoxanthin	0.21 ^d^	0.04, 0.36	0.23 ^c^	0.07, 0.38	0.31 ^c^	0.16, 0.45	0.36 ^c^	0.21, 0.50
Lycopene	0.13	−0.04, 0.27	0.17	−0.01,0.32	0.19 ^d^	0.02, 0.34	0.23 ^c^	0.07, 0.38
Lutein + zeaxanthin	0.12	−0.05, 0.25	0.16	−0.01, 0.31	0.11	−0.01, 0.30	0.14	−0.03,0.27
Vitamin E	0.08	−0.07, 0.24	0.20 ^d^	0.04, 0.36	n/a ^e^			

^a^ Spearman rank correlation using crude intakes and plasma biomarkers; ^b^ Spearman rank correlation using energy-adjusted intakes and plasma biomarkers; ^c^ *P*<0.01; ^d^ *P*<0.05; ^e^ No comparison made between plasma Vitamin E and fruit and vegetables because Vitamin E had more diverse sources.

Quartile agreements between individual nutrient intakes and their respective blood concentrations were in the range of 24%–31% for correctly classified within the same quartile and 62%–72% for correctly classified within the same or adjacent quartile (Table 3). Extremely low quartile agreements were observed for lutein + zeaxanthin. For fruit and vegetable intakes, quartile agreements were similar to those comparing individual carotenoid intakes. However, quartile agreements for fruit and vegetable intakes and β-cryptoxanthin were much higher (>34% within same quartile and >74% within the same/adjacent quartile). 

**Table 3:** Agreement (%) between quartiles of FFQ estimated intakes and biomarkers.

	Individual Nutrient Intakes				Fruit and Vegetable Intakes			
	Crude ^a^		Energy-Adjusted ^b^		Crude ^a^		Energy-Adjusted ^b^	
	Same	Adjacent	Same	Adjacent	Same	Adjacent	Same	Adjacent
α-carotene	30	70	30	72	30	68	32	69
β-carotene	28	69	31	72	29	70	30	72
β-crytoxanthin	30	68	28	71	34	74	40	75
Lycopene	29	68	30	72	34	71	32	74
Lutein + zeaxanthin	24	62	24	65	22	62	21	62
Vitamin E	28	67	28	70	n/a^c^			

^a^ Percentage correctly classified using crude intakes and plasma biomarkers; ^b^ Percentage correctly classified using energy-adjusted intakes and plasma biomarkers; ^c^ No comparison made between plasma Vitamin E and fruit and vegetables because Vitamin E had more diverse sources.

## 4. Discussion

This study determined the relative validity of the Older Australian’s FFQs used in the BMES and HCS by comparing self-reported dietary carotenoid and Vitamin E intakes with more objective plasma biomarkers. Overall, we found that this FFQ performed reasonably well in assessing intakes of carotenoids, Vitamin E, and fruit and vegetables. Although all correlations presented were modest in magnitude (≤0.36), they were comparable to those noted by other validation studies conducted in populations across Australia [10,11] and other countries [12,25,26,27]. More than 68% of individuals were correctly classified within the same or adjacent quartile, based on all nutrients assessed, with the exception of lutein + zeaxanthin. 

We identified two other recent FFQ and biomarker validation studies conducted in Australia [10,11]. One of these studies was the validation study for the commercially available Dietary Questionnaire for Epidemiological Study used in a number of large epidemiological studies, including the Melbourne Collaborative Cohort Study, the Australian Prostate Cancer Family Study, and the Australian Longitudinal Study of Women’s Health [28]. The correlations between dietary and plasma α-carotene and lycopene in our study were only slightly lower compared to these other two studies where correlations for α-carotene ranged between 0.35 and 0.47 and for lycopene ranged between0.19 and 0.28 [10,11]. Our correlations for β-carotene, β-cryptoxanthin and lutein + zeaxanthin were within the ranges reported in the two Australian studies (β-carotene: 0.22–0.28; β-cryptoxanthin: −0.002–0.46; lutein + zeaxanthin: 0.03–0.29).When we compared our results to four other FFQ-biomarker validation studies conducted in the United States of America, similar correlations were observed, althoughβ-cryptoxanthin showed a stronger correlation in the American studies [12,25,26,27]. These studies reported correlations as follows: α-carotene 0.18–0.35, β-carotene 0.25–0.36, β-cryptoxanthin 0.32–0.45, lycopene 0.002–0.37, lutein + zeaxanthin 0.10–0.47. A stronger correlation between energy-adjusted Vitamin E intake and plasma concentration was observed in our study (*r* = 0.20), compared to these other six studies which reported correlations of 0.05–0.07 for Vitamin E [10,11,12,25,26,27].

Plasma levels of carotenoids were significantly correlated with fruit and vegetable intakes except for lutein + zeaxanthin. The observed correlation coefficients were also similar to other studies (α-carotene: 0.23–0.25; β-carotene: 0.13–0.29; β-cryptoxanthin: 0.17–0.35; lycopene: 0.06–0.21; lutein + zeaxanthin: 0.05–0.18) [12,29], indicating that fruit and vegetable intakes are reasonably well measured by the Older Australian’s FFQs and comparable to other FFQs [12,29]. In fact, plasma β-cryptoxanthin and lycopene were more strongly correlated with fruit and vegetable intakes than with individual nutrients. A similar pattern was observed in another study that examined the correlation between plasma carotenoids and fruit and vegetable intakes [12]. Tucker *et al*. (1999) found that correlations were strongest for β-cryptoxanthin followed by lycopene, and the lowest correlation was observed for lutein + zeaxanthin [12]. 

Our study did not identify any important confounding variables. However, the study may have been under-powered for sub-group analyses and there could be other factors not accounted for, such as cholesterol levels in blood, which other studies have adjusted for [10,11,12]. Adjusting for these factors could potentially improve the correlations. 

Quartile agreements between dietary intakes and plasma concentrations further demonstrated that the Older Australian’s FFQs performed well in ranking individuals according to their carotenoid and Vitamin E intakes. As biomarkers are not a measure of absolute intake, the ability to rank individuals according to their consumption is more important [1]. Apart from lutein + zeaxanthin, the percentages of participants correctly classified within the same quartile, and within the same or adjacent quartile for carotenoids and Vitamin E were comparable to those observed in other studies (>25% within same quartile or >65% within same or adjacent quartile) [12,29]. A much lower quartile agreement was observed for lutein + zeaxanthin. Quartile agreement comparing fruit and vegetable intakes rather than individual carotenoid intakes showed similar results, indicating that simply measuring fruit and vegetable intakes provides a reasonable ranking. Subsequent studies examining the effects of nutrition on health outcomes can be confident that this FFQ not only has the ability to accurately capture individual carotenoid intakes but it is also a good measure of fruit and vegetable intakes.

The advantage of this validation method is that the error associated with biomarkers is unlikely to be associated with the error in self-report measures, thus offering an objective measure of nutrient intake [1]. Furthermore, our study methods comply with that of the EURRECCA scoring system, meeting the criteria of a good quality validation study [13]. The strength of this FFQ is that it is developed specifically for an older population and twice validated; firstly against weighed food records in the BMES [3] and now against nutritional biomarkers in the HCS. Validation against weighed food records demonstrated acceptable reproducibility and validity. The current study further demonstrates reasonable validityin reference to nutritional biomarkers, showing that this FFQ is useful in ranking individuals according to their consumption. However, due to the weaker correlation and low quartile agreements for lutein + zeaxanthin, we are less confident of the ability of this FFQ to measure intake of this nutrient.

## 5. Conclusions

In conclusion, results from the current study, together with findings from previous validation against weighed food records, indicate that the Older Australian’s FFQs can reasonably rank individuals according to their consumption of carotenoids (with the exception of lutein + zeaxanthin), Vitamin E, fruit and vegetables. Future studies can use this FFQ to collect dietary data from the older population knowing that it has acceptable validity.
